# Cognitive Performance and Psychological Distress in Breast Cancer Patients at Disease Onset

**DOI:** 10.3389/fpsyg.2019.02584

**Published:** 2019-11-15

**Authors:** Jochen Kaiser, Jörg Dietrich, Miena Amiri, Isa Rüschel, Hazal Akbaba, Nonda Hantke, Klaus Fliessbach, Bianca Senf, Christine Solbach, Christoph Bledowski

**Affiliations:** ^1^Institute of Medical Psychology, Goethe University Frankfurt, Frankfurt am Main, Germany; ^2^Department of Neurology, Massachusetts General Hospital, Harvard Medical School, Boston, MA, United States; ^3^Department of Neurodegenerative Disease and Geriatric Psychiatry, University of Bonn Medical Centre, Bonn, Germany; ^4^German Center for Neurodegenerative Diseases (DZNE), Bonn, Germany; ^5^Department of Psycho-Oncology, University Cancer Center, University Hospital Frankfurt am Main, Frankfurt am Main, Germany; ^6^Senology Unit, Department of Gynecology and Obstetrics, University Hospital Frankfurt am Main, Frankfurt am Main, Germany

**Keywords:** breast cancer, cognitive functions, psychological distress, attention, memory

## Abstract

**Objective:**

Many cancer patients complain about cognitive dysfunction. While cognitive deficits have been attributed to the side effects of chemotherapy, there is evidence for impairment at disease onset, prior to cancer-directed therapy. Further debated issues concern the relationship between self-reported complaints and objective test performance and the role of psychological distress.

**Method:**

We assessed performance on neuropsychological tests of attention and memory and obtained estimates of subjective distress and quality of life in 27 breast cancer patients and 20 healthy controls. Testing in patients took place shortly after the initial diagnosis, but prior to subsequent therapy.

**Results:**

While patients showed elevated distress, cognitive performance differed on a few subtests only. Patients showed slower processing speed and poorer verbal memory than controls. Objective and self-reported cognitive function were unrelated, and psychological distress correlated more strongly with subjective complaints than with neuropsychological test performance.

**Conclusion:**

This study provides further evidence of limited cognitive deficits in cancer patients prior to the onset of adjuvant therapy. Self-reported cognitive deficits seem more closely related to psychological distress than to objective test performance.

## Introduction

Cancer survivors may experience cognitive decline characterized by impaired attention, processing speed, memory or executive functions (Wefel et al., 2004; Bender et al., 2006; Vardy et al., 2007; Dietrich et al., 2008; Lindner et al., 2014; Van Arsdale et al., 2016; Rick et al., 2018). Perceived cognitive deficits can have substantial effects on daily functioning and quality of life (Boykoff et al., 2009). Previous research has attributed cognitive dysfunction to the neurotoxic side effects of chemotherapy (Silberfarb, 1983; van Dam et al., 1998; Schagen et al., 1999). Preclinical studies in rodents have supported this notion by demonstrating the damaging effects of systemically administered chemotherapeutic agents on various cell types of the central nervous system (Dietrich et al., 2006, 2015; Seigers and Fardell, 2011; Monje and Dietrich, 2012). In addition, neuroimaging studies in humans have suggested functional and structural alterations in chemotherapy patients (e.g., Silverman et al., 2007; Deprez et al., 2011, 2013), see reviews by Simo et al. (2013), Kaiser et al. (2014), and Li and Caeyenberghs (2018).

However, other studies have raised a controversial view of chemotherapy-induced cognitive deficits (Hede, 2008; Hermelink, 2015). For example, there is conflicting evidence concerning pre-treatment differences between patients and controls. Several studies have reported cognitive impairment before chemotherapy administration in a certain proportion of breast cancer patients (Hermelink et al., 2007). Affected functions included memory (Lange et al., 2014; Wefel et al., 2004), attention (Jansen et al., 2011), processing speed (Ahles et al., 2008; Hedayati et al., 2011), verbal fluency (Reid-Arndt and Cox, 2012) or visuo-spatial skills (Jansen et al., 2011). In contrast, several other studies found little or no overall cognitive impairment in cancer patients prior to systemic therapy (Jenkins et al., 2006; Schagen et al., 2006; Debess et al., 2010; Mandelblatt et al., 2014; Hermelink et al., 2015).

Further debated issues include the weak association between self-perceived dysfunction and test performance and the contribution of factors such as disease-related distress relative to the effects of chemotherapy. Self-perceived dysfunction is typically more prominent than deficits found in neuropsychological tests (Debess et al., 2010), and both types of measures tend to show only weak correlations. For example, neuropsychological test performance in newly diagnosed breast-cancer patients was unrelated to self-reports of cognitive functions (Cimprich et al., 2005). Similarly, a longitudinal study failed to find associations between chemotherapy-related changes in subjective and objective cognitive measures (Hermelink et al., 2010). In contrast, another large study showed a positive correlation between self-reported cognitive deficits and overall cognitive test performance 1 year after a diagnosis of breast cancer (Hermelink et al., 2017). A recent review found correlations between subjective and objective cognitive impairment in only a third of included studies (Hutchinson et al., 2012). Other studies have reported correlations between subjective and objective memory impairment in subgroups of patients only (e.g., Ganz et al., 2013). In general, subjective complaints seemed more closely associated with measures of psychological distress than with cognitive dysfunction (Cimprich et al., 2005; Hermelink et al., 2010; Biglia et al., 2012; Hutchinson et al., 2012; Yang and Hendrix, 2018).

It is likely that distress contributes to both objective and subjective cognitive dysfunction shortly after a cancer diagnosis (Andreotti et al., 2015). Obviously, being diagnosed with cancer represents a highly stressful event (Hegel et al., 2006; Debess et al., 2009), and stress affects cognitive functioning (Kuhlmann et al., 2005; Schwabe and Wolf, 2013; Arnsten, 2015). In support of this notion, a recent study in breast cancer patients before the start of treatment following initial surgery revealed negative associations between self-reported stress and performance on neuropsychological tests of attention and memory (Reid-Arndt and Cox, 2012). A comparison of cognitive functions before and after chemotherapy showed that decreased performance on some tests correlated with anxiety and depression (Ando-Tanabe et al., 2014). A recent longitudinal study found cancer-induced post-traumatic stress to contribute to subtle cognitive deficits in breast cancer patients (Hermelink et al., 2015, 2017). The association between psychological distress and cognitive performance was also supported by a recent review (Yang and Hendrix, 2018).

In summary, the cause and mechanism of cognitive impairment in cancer patients remains a controversially debated issue. Cognitive complaints prior to systemic treatment have raised doubts about the causative role of chemotherapy and the extent to which treatment contributes to cognitive decline. While there are contradictory findings concerning the associations between subjective and objective cognitive impairments, subjective complaints tended to be more strongly correlated with distress than with performance on neuropsychological tests. These results have given rise to the suggestion that psychological distress caused by the diagnosis of cancer may account for some of the effects previously attributed to chemotherapy. To contribute to this debate, the present study investigated attention and memory in newly diagnosed breast cancer patients and a healthy control group. We focused our investigation on breast cancer because cognitive impairment is of particular relevance to this group of patients due to their high survival rates. Moreover, the vast majority of previous research on cancer-related cognitive dysfunction has been performed in breast cancer patients. We assessed performance shortly after the initial diagnosis and prior to the start of adjuvant therapy. We hypothesized deficits in objective test performance in patients, in addition to self-reported cognitive dysfunction and increased distress levels. Concerning the interrelationships between these variables, we expected stronger correlations between subjective performance and distress measures than between these variables and objective test performance.

## Materials and Methods

### Participants

The study was conducted at the Frankfurt University Hospital, Department of Gynecology and Obstetrics. Adult female patients were recruited either from the inpatient service to which they had been admitted after receiving a diagnosis of breast cancer or from the outpatient clinic. Recruitment took place between August 2015 and June 2017. Exclusion criteria were as follows: previous history of cancer, prior chemotherapy, history of psychiatric or neurologic disease, current use of psychoactive medication, insufficient command of the German language, mental disability, visual impairment or disability of the upper limbs. Healthy female controls were recruited among university staff and family members or friends of the involved researchers between June and August 2016. All participants received a remuneration of €10 per hour. Written informed consent was obtained from all participants. The study was approved by the ethics committee of the University of Frankfurt Medical Faculty.

### Assessment Procedure

A 60-min assessment took place after the diagnosis of breast cancer and prior to the start of chemo- or radiotherapy. Assessments were conducted by advanced-level medical students (MA, IR, HA, and NH) after training and under supervision by the last author (CB), a psychologist with experience in neuropsychological testing.

### Measures

Demographic and clinical data were collected from the participants and from medical records. Pre-morbid verbal intelligence was estimated with a multiple-choice vocabulary test (Mehrfachwahl-Wortschatz-Intelligenztest; Lehrl, 1999).

Cognitive function was assessed with three neuropsychological tests. We used a paper-and-pencil test, the Visual and Verbal Memory Test (VVM; Schellig and Schächtele, 2009) and two computerized tests, the Trail-Making Test, Langensteinbach version (TMT-L; Reitan, 1992) and the Neurocognitive Effects Test (NeuroCogFX; Fliessbach et al., 2006). We focused on subtests tapping either attention or memory. The NeuroCogFX subtests of verbal and figural memory were omitted to keep the duration of testing at a level that was acceptable for patients.

Attention-related measures selected from the NeuroCogFX were simple, go/nogo and inverted go/nogo reaction times and correct responses to the go/nogo and inverted go/nogo subtests. The simple reaction test measured alertness and required participants to hit the space bar of a computer keyboard as quickly as possible whenever a blue circle was presented on the screen. During the go/nogo test, subjects again had to press the space bar for blue circles but had to refrain from responding when a yellow circle was presented. This test thus assessed selective attention. The inverted go/nogo test required the opposite response pattern: responding to the yellow circle and inhibiting the response to the blue circle. This test measured proneness to interference and cognitive flexibility. The TMT-L required participants to connect a set of dots as quickly and accurately as possible. Part A required connecting numbers, while in part B numbers and letters had to be connected in alternation. From the TMT-L, we included processing times and numbers of errors for parts A and B, respectively. The TMT-L thus measured executive function in addition to attention, visual search speed, speed of processing and mental flexibility.

Short-term/working memory was tested using the NeuroCogFX subtests Digit Span and Two–Back Test (hits minus false alarms) and the VVM measures of visual and verbal memory. The visual part of the VVM required the reproduction of a path on a street map, whereas the verbal part tested the recall of details from a written text. Recall was first tested immediately (within approximately 5 min) after learning and then again after a delay of about 30 min.

Anxiety and depression were assessed with the German version of the Hospital Anxiety and Depression Scale (HADS; Snaith and Zigmond, 1986). Quality of life was measured with the cancer-specific European Organization for Research and Treatment of Cancer Quality of Life Questionnaire C30, version 3.0 (EORTC-QLQ-C30; Aaronson et al., 1993). We included the global health status/quality of life scale and the summary scores across the functional and symptom scales/items, respectively, into the statistical analysis. The functional scales comprise subscales in the following domains: physical abilities/fitness, role functioning (job, leisure activities), cognitive, emotional and social functioning. A high score for the functional scales represents a high/healthy level of functioning. The symptom scales include subscales/items measuring physical symptoms like fatigue, nausea, vomiting, insomnia, appetite loss, but also financial difficulties. A high score for the symptom scales represents a high level of symptomatology/problems. The cognitive functions (CF) subscale of the EORTC-QLQ-C30 was used to estimate subjective cognitive functioning. It comprises two items tapping concentration and memory problems. Here higher scores represented a higher level of functioning.

### Statistical Analysis

As more than half of the cognitive variables were not normally distributed, differences between patients and control participants on demographic, cognitive and distress-related variable were evaluated with non-parametric tests (Mann–Whitney-*U* test for independent samples) or Pearson’s chi-squared tests. Effect sizes (Cohen’s *d*) were calculated for variables with statistically significant (*p* < 0.05, uncorrected) group differences.

To explore the relationships between neuropsychological test performance and distress, we calculated Spearman-rho rank correlation coefficients between cognitive measures showing group differences on the one hand, and anxiety, depression and quality of life variables on the other hand. The relationship between subjective and objective measures of cognitive functioning was assessed by correlating the same cognitive measures with the “cognitive functions” subscale of the EORTC-QLQ-C30. Finally, we assessed correlations between the “cognitive functions” subscale of the EORTC-QLQ-C30 and measures of psychological distress (anxiety and depression).

## Results

### Demographic and Treatment Characteristics

Twenty-seven patients and 20 healthy controls participated in the study. Recruitment proved difficult because of the short time window between diagnosis and the onset of systemic therapy and the patients’ limited willingness to take part in the study at that particular point in time. The participants’ demographic characteristics are provided in Table 1. Patients and controls did not differ in age, body mass index, number of children, relationship status, education level, employment status, household income or verbal intelligence. The only difference was found for physical activity, where a smaller proportion of patients (57.8%) than controls (90%) indicated spending at least 1 h per week with physical exercise activities. Most patients had undergone surgery (sentinel node biopsy or breast-conserving surgery) prior to testing. Surgery had taken place within 1 week (54% of patients), within 1 month (23%) or more than 1 month (11.5%) prior to testing. The remaining 11.5% of patients underwent surgery 1 day after testing.

**TABLE 1:** Demographic characteristics.

**Characteristic**	**No. of patients (%) (*n* = 27)**	**No. of controls (%) (*n* = 20)**	***P***
** Age, *y***			
Mean (SD)	53.2 (9.0)	49.0 (11.2)	0.426^†^
Range	33–70	28–67	
** Body mass index**			
Mean (SD)	25.8 (6.3)	23.8 (3.0)	0.397^†^
No. of children, mean (SD)	1.2 (1.04)	1.60 (1.00)	0.162^†^
Living without a partner	9 (33.3)	6 (30.0)	0.808^§^
**Educational level^‡^**			0.613^§^
Low	4 (14.8)	3 (15.0)	
Medium	10 (37.0)	5 (25.0)	
High	4 (14.8)	6 (30.0)	
University degree	9 (33.3)	6 (30.0)	
Employment status			0.095^§^
Unemployed	9 (33.3)	3 (15.0)	
Part-time employment	9 (33.3)	13 (65.0)	
Full-time employment	9 (33.3)	4 (20.0)	
**Household income (€/month)^¶^**			0.923^§^
<2000	7(29.2)	5 (27.8)	
2000–4000	12 (50.0)	10 (55.6)	
>4000	5 (20.8)	3 (16.7)	
**Exercise activities (h/week)^¶¶^**			0.017^§^
<1	11 (42.3)	2 (10.0)	
1–3	8 (30.8)	12 (60.0)	
3–5	6 (23.1)	2 (10.0)	
>5	1 (3.8)	4 (20.0)	
** Premorbid intelligence**			
Mean IQ (SD)	106.1 (11.8)	107.0 (11.9)	0.711^†^

*H = hours; SD = standard deviation; y = years. ^†^Mann–Whitney-*U* test for independent samples. ^‡^Low, Hauptschulabschluss; medium, Mittlere Reife; high, Fachhochschulreife or Abitur, corresponding to 9, 10, and 13 years of schooling, respectively. ^§^ Pearson’s chi-squared test ^¶^ Three patients and two control subjects did not indicate their household income. ^¶¶^ One patient did not indicate her exercise activity.*

### Cognitive Function

When applying an uncorrected *p*-value of 0.05, neuropsychological test performance differed between patients and controls on only three out of the 15 measures. Among the attention-related measures, the only group difference was found for the inverse go/nogo task of the NeuroCogFX test, where patients had longer reaction times than controls (Table 2). In contrast, there were no reaction-time differences for the simple reaction or the go/nogo tasks of the NeuroCogFX, nor were there differences in accuracy for the go/nogo or inverted go/nogo tests. Similarly, none of the measures derived from the TMT-L differed between patients and controls. Considering the memory-related measures, group differences were found for both immediate and delayed recall of the VVM verbal memory test (Table 3). In contrast, patients and controls did not differ on the visual memory tests of the VVM. This suggested that the deficit was specific to verbal memory. No differences were found for any of the memory measures of the NeuroCogFX (digit span or two-back test). Effect sizes for both attention- and memory-related group differences ranged between 0.76–0.79, indicating medium-size effects.

**TABLE 2:** Attention-related cognitive tests.

**Cognitive measure**	**Patients mean (SD)**	***n***	**Controls mean (SD)**	***n***	**Power**	**Cohen’s *d***	***p*^†^**
**NeuroCogFX**							
Simple RT, ms	347 (48)	26	325 (38)	20	0.69	0.50	0.118
Go/nogo RT, ms	456 (60)	26	429 (52)	20	0.69	0.48	0.135
Inverse go/nogo RT, ms	455 (51)	26	419 (42)	20	0.66	0.76	0.019
Go/nogo correct responses	9.38 (0.50)	26	9.35 (0.49)	20	0.86	0.06	0.812
Inverse go/nogo corr. resp.	9.23 (0.51)	26	9.50 (0.51)	20	0.67	0.53	0.091
**Trail-making test**							
Completion time part A, s	21.6 (5.3)	27	23.1 (5.6)	20	0.67	0.28	0.302
Completion time part B, s	39.6 (16.4)	27	38.5 (13.9)	20	0.98	0.08	0.957
Errors part A	0.15 (0.46)	27	0.05 (0.22)	20	0.76	0.26	0.450
Errors part B	2.22 (3.26)	27	1.05 (1.47)	20	0.91	0.44	0.430

*Corr. resp. = correct responses; ms = milliseconds; NeuroCogFX = neurocognitive effects test; RT = reaction time; s = seconds; SD = standard deviation, ^†^Mann–Whitney-*U* test for independent samples.*

**TABLE 3:** Memory-related cognitive tests.

**Cognitive measure**	**Patients mean (SD)**	***n***	**Controls mean (SD)**	***n***	**Power**	**Cohen’s *d***	***p*^†^**
**NeuroCogFX**							
Digit span	5.93 (1.11)	27	5.55 (1.00)	20	0.72	0.36	0.260
Two-back test (hits minus FA)	7.88 (2.25)	26	7.80 (2.86)	20	0.93	0.03	0.856
**VVM**							
Visual immediate recall	21.2 (7.4)	27	21.6 (7.1)	20	0.87	0.06	0.821
Verbal immediate recall	10.0 (4.7)	27	13.6 (4.5)	20	0.74	0.78	0.026
Visual delayed recall	19.9 (6.9)	27	20.2 (6.8)	20	0.84	0.04	0.804
Verbal delayed recall	9.6 (4.4)	27	13.1 (4.5)	20	0.71	0.79	0.020

*FA = false alarms; NeuroCogFX = Neurocognitive effects test; SD = standard deviation; VVM = Visual and Verbal Memory Test. ^†^Mann–Whitney-*U* test for independent samples.*

To explore whether the observed effects were related to differences in physical activity between patients and controls, we compared cognitive performance within the patient group between patients who were physically inactive (<1 h of exercise activity per week, *n* = 11) and those who were physically active (>1 h of physical exercise per week, *n* = 15). There were no differences for either inverse go/nogo reaction time (*p* = 0.698), verbal memory performance at immediate recall (*p* = 0.482) or delayed recall (*p* = 0.434).

Furthermore, we explored within the patient group whether the interval between surgery and testing affected cognitive performance on those tests where patients and controls showed differences. There were weak to moderate correlations that did not reach significance between the number of days after surgery and test performance for the three variables: inverse go/nogo reaction time (*r* = −0.346, *p* = 0.114), verbal memory: immediate recall (*r* = 0.305, *p* = 0.157), and verbal memory: delayed recall (*r* = 0.218, *p* = 0.317).

### Distress and Quality of Life

As shown in Table 4, patients reported higher levels of anxiety and depression than healthy controls. On average, scores for both variables were in the subclinical range, i.e., below a raw score of 8. Applying this criterion, clinical levels of anxiety were reported by 37% of patients compared with 10% of controls, while clinical levels of depression were found in only 7% of patients and in none of the control participants. Patients also reported a poorer quality of life than controls. Effect sizes ranged from 0.69 to 1.64, indicating medium to strong effects. Exploring the effects of temporal proximity to surgery, no significant correlations were observed between days after surgery and anxiety (*r* = −0.053, *p* = 0.811), depression (*r* = 0.120, *p* = 0.584), global health status (*r* = −0.190, *p* = 0.386) or subjective cognitive functions (*r* = 0.194, *p* = 0.374).

**TABLE 4:** Psychological distress and quality of life.

**Characteristic**	**Patients mean (SD)**	***n***	**Controls mean (SD)**	***n***	**Power**	**Cohen’s *d***	***p*^†^**
**HADS**							
Anxiety	7.1 (3.7)	27	3.3 (3.1)	20	0.67	1.10	0.001
Depression	4.0 (2.4)	27	1.6 (1.8)	20	0.68	1.11	0.001
**EORTC-QLQ-C30**							
Global health status/QoL	57.1 (22.4)	27	79.6 (17.0)	20	0.68	1.11	<0.001
Functional scales	65.0 (16.7)	27	87.7 (8.6)	20	0.99	1.64	<0.001
Symptom scales	22.3 (15.8)	27	7.6 (7.6)	20	0.70	1.13	0.001
Cognitive functions (CF)	69.8 (23.6)	27	82.5 (17.5)	20	0.68	0.60	0.061

*EORTC-QLQ-C30 = European Organization for Research and Treatment of Cancer Quality of Life Questionnaire; HADS = Hospital Anxiety and Depression Scale; QoL = quality of life; SD = standard deviation. ^†^Mann–Whitney-*U* test for independent samples.*

### Correlations Between Cognitive Tests, Self-Reported Function and Distress

To explore the interrelationships between the different types of variables, we calculated correlations between those measures for which group differences were found. Data from all participants (patients and controls) were included. Cognitive function was mostly unrelated to measures of distress or self-reported cognitive complaints (Table 5). The only correlation that reached significance at *p* < 0.05 (uncorrected) was found between verbal delayed recall performance and anxiety (*p* = 0.041): higher anxiety scores were associated with poorer memory performance. When patients and controls were assessed separately, correlations were weaker and non-significant (patients: *r* = −0.169; controls: *r* = −0.127; no significant difference between groups). This raises the possibility that the correlation across groups was attributable to group differences in anxiety and verbal delayed recall memory.

**TABLE 5:** Correlations^†^ between cognitive function, distress and self-reported cognitive functions.

	**Inverse go/nogo RT (*n* = 46)**	**Verbal memory (IR) (*n* = 47)**	**Verbal memory (DR) (*n* = 47)**
**HADS**			
Anxiety	0.183	–0.275	−0.299^∗^
Depression	0.174	–0.257	–0.241
**EORTC-QLQ-C30**			
Global health status/QoL	–0.266	0.195	0.205
Functional scales	–0.224	0.165	0.165
Symptom scales	0.183	–0.128	–0.142
Cognitive functions subscale	–0.109	0.254	0.233

*DR = delayed recall; EORTC-QLQ-C30 = European Organization for Research and Treatment of Cancer Quality of Life Questionnaire; HADS = Hospital anxiety and depression scale; IR = immediate recall; QoL = quality of life; RT = reaction time. ^†^Spearman-rho rank correlation coefficient. ^∗^*p* < 0.05.*

In contrast, in the entire sample (patients and controls), distress and quality of life measures were highly intercorrelated with correlation coefficients between 0.36 and 0.66 (most *p* < 0.001) (Table 6). Self-reported cognitive functions were also correlated with both anxiety (*p* < 0.001) and depression (*p* = 0.018), reflecting the close link between subjective complaints and psychological distress. The correlations between anxiety and self-reported cognitive function reached significance within each group (patients: *r* = −0.393, *p* = 0.043; controls: *r* = −0.467, *p* = 0.038; no significant difference between groups), suggesting that this association was not due to the variance between groups. For depression, the correlations with self-reported cognitive function were weaker and non-significant within each group (patients: *r* = −0.241; controls: *r* = −0.262). Again, there was no significant difference between groups.

**TABLE 6:** Correlations^†^ between measures of distress and quality of life.

	**EORTC-QLQ-C30 Global health/QoL (*n* = 47)**	**EORTC-QLQ-C30 Functional scales (*n* = 47)**	**EORTC-QLQ-C30 Symptom scales (*n* = 47)**	**EORTC-QLQ-C30 Cognitive functions (*n* = 47)**
**HADS**				
Anxiety	−0.360^∗^	–0.568^∗∗∗^	0.473^∗∗^	–0.520^∗∗∗^
Depression	–0.666^∗∗∗^	–0.594^∗∗∗^	–0.596^∗∗∗^	−0.353^∗^

*EORTC-QLQ-C30 = European Organization for Research and Treatment of Cancer Quality of Life Questionnaire; HADS = Hospital Anxiety and Depression Scale; QoL = quality of life. ^†^Spearman-rho rank correlation coefficient. ^∗^*p* < 0.05, ^∗∗^*p* < 0.01, ^∗∗∗^*p* < 0.001.*

## Discussion

The present study assessed cognitive function with a focus on attention and memory, psychological distress and quality of life in breast cancer patients prior to adjuvant treatment and in healthy controls. Patients and controls differed on a small subset of tests. Patients had longer reaction times on the inverted go/nogo test and showed poorer verbal memory performance at both immediate and delayed recall. In contrast, there were pronounced group differences in anxiety, depression and quality of life. While these distress measures were intercorrelated and also associated with self-reported cognitive functioning, correlations with cognitive test performance were mostly absent.

### Cognitive Function Prior to Adjuvant Therapy

Among the different attention–related tests, the only group difference was found for reaction times on the inverted go/nogo task of the NeuroCogFX test (Table 2). In contrast, there were no group differences on any of the other attention tests including the other choice-reaction subtests of the NeuroCoGFX or the TMT. Slower response speed has been reported repeatedly in breast cancer patients prior to adjuvant therapy (Ahles et al., 2008) and even prior to a confirmed diagnosis (Hedayati et al., 2011). The sensitivity of go/nogo tasks to cognitive impairment has been demonstrated also by two recent studies that have observed increased error rates both immediately after a cancer diagnosis (Hermelink et al., 2015) and in cancer survivors (Wirkner et al., 2017). Here, we found poorer performance in patients prior to adjuvant therapy only for the more challenging, inverted version of the go/nogo task where the stimulus-response assignment was swapped compared with the preceding task. Interestingly, correct response rates did not differ between patients and controls. Apparently, patients achieved their high level of accuracy by responding more slowly, possibly reflecting increased susceptibility to interference and reduced cognitive flexibility.

Considering memory–related tests, patients showed poorer performance than controls for the verbal memory test of the VVM (Table 3). This difference was present both for recall after five and after about 30 min. The extremely high correlation between both recall intervals of *r* = 0.978 (*p* < 0.001) suggests that these measures can hardly be considered to reflect different processes. In contrast, patients and controls did not differ on the digit-span or two-back tests of the NeuroCogFX or on the visual memory subtests of the VVM. The present finding of memory impairment in newly diagnosed breast cancer patients is consistent with several studies that have reported verbal memory deficits in cancer patients both prior to therapy (Lange et al., 2014) and after treatment with chemotherapy (Von Ah et al., 2009; Lindner et al., 2014; Wirkner et al., 2017; Li and Caeyenberghs, 2018).

Prior to testing, the majority of patients in the present study had undergone surgery, which can have transient deleterious effects on cognitive function (Johnson et al., 2002). Accordingly, patients tested shortly after surgery showed a slightly poorer cognitive performance than those with a longer delay between surgery and testing. However, this association did not reach statistical significance in the present sample. It is nevertheless conceivable that the fact that about half of our patients were tested shortly after surgery might have contributed to the present findings. Future research is needed to assess this question more systematically.

In summary we found slower reaction times in the inverted go/nogo task and poorer verbal memory in cancer patients prior to adjuvant therapy. However, these results have to be treated with caution considering both the limited sample size and the fact that they would not have withstood a correction for multiple testing. This means that if cognitive impairment is present at all in newly diagnosed breast cancer patients, effects are limited to selected subtests. Processing speed in cognitively demanding situations and memory for names, numbers and facts in a written text might be particularly vulnerable to the effects of a cancer diagnosis. As impairments of both types of functions are relevant to many everyday activities, they are likely to contribute to the subjective experience of cancer-related cognitive decline.

### Relationships Between Cognitive Function, Distress and Self-Reported Complaints

As expected, patients reported elevated, yet subclinical levels of anxiety and depression as measured with the HADS (Table 4). Taken together with the poorer quality of life in patients compared to controls, these findings confirm that a cancer diagnosis is a highly stressful event. In addition to receiving the diagnosis, the vast majority of patients had undergone surgery prior to testing and all of them were tested during acute care, which is known to be associated with high distress levels (Senf et al., 2010).

Psychological distress in the present sample was mostly uncorrelated with neuropsychological test performance (Table 5). Only a single correlation between anxiety and verbal delayed recall memory reached significance at a liberal, uncorrected criterion of *p* < 0.05. This is consistent with previous studies that found no correlations between variables of psychological well-being and objective tests of cognitive function during the initial phase of illness (Cimprich, 1992; Lehto and Cimprich, 1999; Jenkins et al., 2006) or after therapy (Biglia et al., 2012). In contrast, investigations with larger samples provided some evidence for associations between various measures of distress and both verbal memory and processing speed (Menning et al., 2015), and between go/nogo errors and symptoms of post-traumatic stress disorder (Hermelink et al., 2015). Similar to impairments on cognitive tests, correlations between emotional and cognitive measures in newly diagnosed patients may be subtle and therefore hard to detect with a small sample size like in the present study. Alternatively, disease-related factors other than psychological distress may contribute to cognitive impairment. For example, neuroinflammatory processes accompanying tumor growth have been found to affect both emotional well-being and cognition (Patel et al., 2015; Schrepf et al., 2015). According to this view, changes should be present prior to a diagnosis of cancer. This issue requires further investigation.

Cognitive performance was also unrelated to self-reported cognitive complaints as assessed with the cognitive functions subscale of the EORTC-QLQ C-30 scale (Table 5). While some investigations have reported associations between subjective cognitive complaints and memory test performance (Ganz et al., 2013; Lange et al., 2014), others found no or only weak correlations between both types of measures (Cimprich, 1992; Cimprich et al., 2005; Schilder et al., 2012). In contrast, self-reported cognitive problems showed highly significant correlations with anxiety, depression and quality of life measures (Table 6). While the contribution of a common-method bias leading to increased correlations between measures obtained with the same method (i.e., self-report) cannot be excluded (Podsakoff et al., 2003), this finding is consistent with numerous studies demonstrating close associations between perceived cognitive impairment and distress-related measures including anxiety, depression, hyperarousal and fatigue (Lehto and Cimprich, 1999; Cimprich et al., 2005; Biglia et al., 2012; Schilder et al., 2012; Li et al., 2015). The present data thus support the notion that cancer-related subjective complaints reflect psychological distress rather than objectively measured cognitive function (Yang and Hendrix, 2018). Again, this conclusion has to be drawn with caution given the limited sample size.

## Conclusion

The present results are in keeping with recent findings of research on cancer-related cognitive impairment at disease onset. Cognitive deficits were found only for a small number of subtests tapping verbal memory and processing speed. In contrast, patients differed robustly from controls both on measures of psychological distress and quality of life. Subjective impairment was unrelated to objective test performance but closely correlated with distress. The present study thus suggests that cognitive dysfunction in newly diagnosed cancer patients may be rather subtle and hard to detect with neuropsychological tests, and that psychological distress plays an important role in perceived cognitive decline.

## Data Availability Statement

The datasets generated for this study are available on request to the corresponding author.

## Ethics Statement

The studies involving human participants were reviewed and approved by the Ethik-Kommission des Fachbereichs Medizin, Universitätsklinikum der Goethe-Universität, Theodor-Stern-Kai 7, 60596 Frankfurt am Main. The patients/participants provided their written informed consent to participate in this study.

## Author Contributions

JK, JD, BS, CS, and CB conceived and designed the study. MA, IR, HA, and NH collected the data. JK and CB analyzed the data. JK, JD, KF, and CB interpreted the data. JK, JD, and CB prepared the manuscript. All authors contributed to the critical discussions.

## Conflict of Interest

The authors declare that the research was conducted in the absence of any commercial or financial relationships that could be construed as a potential conflict of interest.
